# Smart Lobbying for Minimum Nurse Staffing Ratios in Spain: Not Just Numbers

**DOI:** 10.1177/1527154420923753

**Published:** 2020-05-05

**Authors:** Enrique Castro-Sánchez, Azucena Santillán-García

**Affiliations:** Lead Academic Research Nurse, Imperial College London; Staff Nurse Cardiology, University Hospital of Burgos

In her farewell December 2019 editorial, Dr. Cohen highlighted pending nursing policy priorities including the balancing of resources among multiple micro-to-macro health policy levels (Cohen, 2019). National legislation for minimum nurse/patient ratios remains among these policy needs in many countries, including Spain. An intense nursing union-driven lobbying campaign has just garnered more than 500,000 signatures, warranting that a legislative motion is tabled for debate at parliament. However, while we agree that current Spanish nursing workforce is unfit for purpose, we reflect on structural challenges demanding consideration before engaging in political and policy lobbying.

Undoubtedly, the shortage of nurses in health and social care systems worldwide is one of the most pressing challenges to achieve goals such as universal health care coverage. By now, negative outcomes of inadequate nursing staffing such as mortality and “missed care” for patients and services have been widely reported by methodologically robust studies (Aiken et al., 2011; NIHR Dissemination Centre, 2019). Further, this evidence emphasized how essential it is for an adequate number of nurses to be coupled with excellent human resources and team management, underpinned by a nurturing professional and institutional climate.

Unfortunately, those aspects seem ignored by the Spanish lobbyists. They aspire for the Spanish nursing workforce to be similar in size to economically comparable countries. This perspective risks disregarding the needs of citizens and patients, as well as sociocultural idiosyncrasies, or even the notion of what a “nurse” is on each country (Rafferty et al., 2019). Linked to that, emerging evidence points to nursing workload and volume of care activities as important if not more than the crude number of patients per nurse (Margadant et al., 2020). Thus, any debate about specific ratios must be complemented by a narrative on the complexity of care required, including optimal skill- and competency-mix of different nurse practitioners (i.e., generalist, specialist, advanced nurses, etc.).

However, these calls for increased staffing are yet to determine which health and social care needs must be met, at what level, and by which models of nursing service delivery or roles. The mere transplantation of staffing levels across health systems, in itself, would not address the question of “how many nurses are needed to meet the care needs of a given population, within a certain professional and cultural framework?” The gap in the evidence is even larger in relation to areas such as family and community care, nursing and long-term care facilities, or management and education. With regard to education, by the way, there has not been any debate about how the current educational framework would be able to accommodate enough nursing lecturers or secure practice placements for the required professionals.

Finally, we cannot ignore the challenges of optimal workforce management, further compounded by the prevailing recruitment and hiring model in Spain. This model outputs thousands of nurses each year who are, effectively, sandwiched between national selection processes held with luck every few years, and short-term contracts in services for which nurses may not have any experience, expertise, or interest. Unless this employment model offers some security, achieving the proposed nurse ratios may do little to improve working practices and care outputs sustainably. We may even witness a scenario where the desired number of nurses is in place but their fragmented employment around the system may negate the patient benefits reported in the studies.

To sum up, as evidence-informed practitioners, we welcome nurse staffing improvements across Spain. But, equally, we are uncomfortable with lobbying campaigns which seem too willing to ignore the care needs that Spanish patients and citizens may have and thus the optimal number and range of nurses required to meet those needs.

## References

[bibr1-1527154420923753] Aiken L. Cimiotti J. Sloane D. Smith H. Flynn L. Neff D. (2011). Effects of nurse staffing and nurse education on patient deaths in hospitals with different nurse work environments. Medical Care, 49(12), 1047–1053. 21945978 10.1097/MLR.0b013e3182330b6e PMC3217062

[bibr2-1527154420923753] Cohen S. S. (2019). Transitions, reflections, and visions for the future. Policy, Politics, & Nursing Practice, 20(4), 179–180. 10.1177/1527154419894828 31825752

[bibr3-1527154420923753] Margadant C. Wortel S. Hoogendoorn M. Bosman R. Spijkstra J. Brinkman S. de Keizer N. (2020). The nursing activities score per nurse ratio is associated with in-hospital mortality, whereas the patients per nurse ratio is not. Critical Care Medicine, 48(1), 3–9. 10.1097/CCM.0000000000004005 31841450

[bibr4-1527154420923753] NIHR Dissemination Centre. (2019). *Staffing on wards* (1st ed.). 10.3310/themedreview-03553

[bibr5-1527154420923753] Rafferty A. Busse R. Zander-Jentsch B. Sermeus W. Bruyneel L. (2019). Strengthening health systems through nursing. World Health Organization, European Observatory on Health Systems and Policies http://www.euro.who.int/en/about-us/partners/observatory/publications/studies/strengthening-health-systems-through-nursing-evidence-from-14-european-countries-2019 31465161

